# Supplementary material for: Cloud BioLinux: pre-configured and on-demand bioinformatics computing for the genomics community
Source: BMC Bioinformatics. 2012 Mar 19;13:42. doi: 10.1186/1471-2105-13-42 (PMC3372431; doi:10.1186/1471-2105-13-42)
Supplement: Additional file 1 — Supplementary 1 Cloud BioLinux software documentation in the form of a mini, self-contained website. Users need to download and uncompress the .zip file, and open through a web browser the "index.html" file available on the main directory. (ZIP 1823 kb). [file 1471-2105-13-42-S1.ZIP › Cloud-BioLinux-Package-Documentation/docs/arb.html]

Bio-Linux Software Documentation Pages

Back to search form

## arb

|  |  |
| --- | --- |
| Name | arb |
| Description | **ARB** is a graphically oriented package comprising various tools for sequence database handling and data analysis. A central database of processed (aligned) sequences and any type of additional data linked to the respective sequence entries is structured according to phylogeny or other user defined criteria.  ARB was primarily been developed for ribosomal ribonucleic acid (rRNA) sequences, and therefore contains special tools for alignment and analysis of these structures. However, other molecular sequence data can also be handled. Protein gene sequences and predicted protein primary structures can be stored in the same database.  **ARB** is designed for a graphical user interface. Program control and data display are available in a hierarchical set of windows and subwindows. The majority of operations can be controlled using the mouse for moving the pointer and the left mouse button for initiating and performing operations.  The following tools are available on ARB:  **ARB\_DB**   - A central database of (aligned homologous) sequences and additional information, taken from public databases or supplied by the user, is stored in a (binary or ASCII) file (\*.arb). - The database can be structured according to phylogeny or other user-defined criteria. - Tools for text-oriented database searching are integrated.  **ARB\_NT**   - Phylogenetic trees derived from the data or imported from other sources are displayed within the main window. Different tree topologies, complete trees, and subtrees can be stored and used for walking through the database. Database entries can be shown with the tree o­n the screen or in separate windows. Trees can be used to define subsets of data for display or analysis by other ARB tools. - Publication-ready trees can be produced by shaping the displayed tree topology and printing or exporting the tree to foreign software (TREETOOL [], XFIG []).  **ARB\_EDIT**  An editor for the display of sequences and sequence-associated data (masks and filters, consensus sequences, higher-order structure) and basic editing functions is available. This tool allows manual entering of new sequences (with a customized keyboard, if desired); manual modification of alignments; search and replacement of sequence stretches; and printout of data. Predicted higher-order structure is automatically checked according to a user-provided mask, and may be displayed with the sequences by user-definable symbols.  **ARB\_ALIGN**  The ARB tool for automated sequence alignment searches for the most similar sequences in the database and inserts the new sequence into an existing alignment according to primary and higher-order structural similarity.  **ARB\_IMPORT/EXPORT**  ARB modules as well as integrated foreign software (GDE [1], READSEQ [2], CONVERTALIGN [3]) can be used for import and export of (subsets of) data in different formats, and for database merging.  **ARB\_PROTECTION**  Up to six hierarchical protection levels can be individually asigned to database entries to prevent unintended modification or loss of data.  **ARB\_NAMES**  Unique identifiers are automatically generated for the individual entries and stored with the database. This prevents multiple entries of the same data, and assignment of identical names to different data.  **ARB\_PHYL**  ARB tools and integrated foreign software (PHYLIP [4], DE SOETE [1], fastDNAml [4]) allow calculation of similarity/distance matrices, conservation profiles, selection masks and phylogentic tree reconstruction using different treeing approaches.  **ARB\_PROBE**  Species- and group-specific probes are designed and checked by searching the complete database for unique sequence stretches. Potential probe or target sites are ranked by user-supplied criteria for mismatch weighing.  An Example file can be found in this directory of your arb installation "/usr/software/sampledata/arb\_demo\_data"  A PDF manual can be found here http://www.arb-home.de  **References:**  Getting started notes [Pdf]    Design and Evaluation of 16S rRNA-Targeted Oligonucleotide Probes for Fluorescence In Situ Hybridization [Entrez]    ARB: a software environment for sequence data. Nucleic Acids Research 32(4):1363-1371. [Entrez]    Fluorescence in situ hybridization with rRNA-targeted oligonucleotide probes (PDF from J.Pernthaler, F.O.GlЖckner, W.SchЖnhuber, R.Amann) [Pdf] |
| Homepage | http://www.arb-home.de/ |
| Remote Documentation | http://www.arb-home.de/documentation.html |

|  |
| --- |
| Please see Remote Documentation |
